# Thymoquinone (TQ) inhibits the replication of intracellular *Mycobacterium tuberculosis* in macrophages and modulates nitric oxide production

**DOI:** 10.1186/s12906-017-1786-0

**Published:** 2017-05-25

**Authors:** Hafij Al Mahmud, Hoonhee Seo, Sukyung Kim, Md Imtiazul Islam, Kung-Woo Nam, Hyun-Deuk Cho, Ho-Yeon Song

**Affiliations:** 1 Department of Microbiology and Immunology, School of Medicine, Soonchunhyang University, Cheonan, Chungnam, 31151 Korea; 2 Department of Life Science and Biotechnology, School of Life Sciences, Soonchunhyang University, Asan, Chungnam, 31538 Korea; 3 Departments of Pathology, Soonchunhyang University Cheonan Hospital, Soonchunhyang University College of Medicine, Cheonan, Korea

**Keywords:** *Mycobacterium tuberculosis*, Xdr-Tb, Intracellular killing, Nitric oxide, Thymoquinone

## Abstract

**Background:**

Human tuberculosis, which is caused by the pathogen *Mycobacterium tuberculosis*, remains a major public health concern. Increasing drug resistance poses a threat of disease resurgence and continues to cause considerable mortality worldwide, which necessitates the development of new drugs with improved efficacy. Thymoquinone (TQ), an essential compound of *Nigella sativa*, was previously reported as an active anti-tuberculosis agent.

**Methods:**

In this study, the effects of TQ on intracellular mycobacterial replication are examined in macrophages. In addition, its effect on mycobacteria-induced NO production and pro-inflammatory responses were investigated in *Mycobacterium tuberculosis* (MTB)-infected Type II human alveolar and human myeloid cell lines.

**Results:**

TQ at concentrations ranging from 12.5 to 25 μg/mL and 6.25 to 12.5 μg/mL reduced intracellular *M. tuberculosis* H37Rv and extensively drug-resistant tuberculosis (XDR-TB) 72 h post-infection in RAW 264.7 cells. TQ treatment also produced a concentration-dependent reduction in nitric oxide production in both H37Rv and XDR-TB infected RAW 264.7 cells. Furthermore, TQ reduced the expression of inducible nitric oxide synthase (iNOS) and pro-inflammatory molecules such as tumor necrosis factor-alpha (TNF-α) and interlukin-6 (IL-6) in H37Rv-infected cells and eventually reduced pathogen-derived stress in host cells.

**Conclusions:**

TQ inhibits intracellular H37Rv and XDR-TB replication and MTB-induced production of NO and pro-inflammatory molecules. Therefore, along with its anti-inflammatory effects, TQ represents a prospective treatment option to combat *Mycobacterium tuberculosis* infection.

## Background

Tuberculosis (TB) still accounts for millions of cases of active disease and deaths worldwide and remains a global health emergency. In 2015, the World Health Organization estimated that there were 10.4 million new (incident) TB cases worldwide, of which people with HIV accounted for 1.2 million (11%) [1]. The emergence of multidrug-resistant tuberculosis (MDR-TB) and extremely drug-resistant tuberculosis (XDR-TB) is a major threat to global tuberculosis control, which is continually driven by inappropriate tuberculosis treatment [2, 3]. Therefore, the current 40-year-old treatment of tuberculosis with its cytotoxicity and complexity necessitates a new anti-tuberculosis agent with improved efficacy and safety [3].

*Mycobacterium tuberculosis* can enhance the production of nitric oxide (NO) in infected cells [4]. Nitric oxide a small reactive nitrogen intermediate (RNI) that is produced from arginine by an enzymatic reaction catalyzed by the enzyme nitric oxide synthase (NOS) [5] in response to different cytokines. The production of RNI in host cells is considered an antimicrobial agent against intracellular microorganisms [6]. However, there is a point of controversy: host-derived stress such as that originating from reactive oxygen species (ROS) and RNI induces drug tolerance in *Mycobacterium tuberculosis* [7]*.* Furthermore, excessive production of NO has cytotoxic effects and leads to nuclear DNA damage, which could ultimately bring about cell death [5].

Thymoquinone (TQ; 2-isopropyl-5-methyl-1, 4-ben-zoquinone), the main active component of the essential oil of *Nigella sativa* (Ranunculaceae) seeds, has antibacterial and antitubercular activities [8]. Moreover, TQ inhibits *E. coli*-induced NO production in sepsis [9], and its anti-inflammatory and anti-cancer effects have been reported in both in vivo and in vitro models [10] [11].

This study was carried out to demonstrate the intracellular killing effect of TQ in H37Rv and XDR-TB *M. tuberculosis* infected macrophages*.* In addition, we investigated the effect of TQ on *M. tuberculosis-*induced pro-inflammatory cytokines and NO expression. Our results show that TQ (i) inhibits the replication of intracellular H37Rv and XDR-TB *M. tuberculosis* in mouse macrophage RAW 264.7 cells and (ii) reduces the production of MTB-induced pro-inflammatory cytokines (IL-6 and TNF-α) and NO in human type II alveolar epithelial cells (A549) and (phorbol-12-myristate-13-acetate) PMA-induced human macrophage THP-1 cells in vitro.

## Methods

### Bacterial strains and growth conditions

*M. tuberculosis* strain H37Rv (American Type Culture Collection; ATCC 35835) and XDR-TB-TB (Korean Microorganism Resource Center; KMRC 00203–00197) were used as reference strains. The recombinant strain of *M. tuberculosis* H37Ra expressing green fluorescent protein (H37Ra-GFP) bears an integrative plasmid (pFPCA1) constructed via methods described by Changsen et al. [12]. The pFPCA1 plasmid was kindly provided by Dr. Palittapongarnpim, and electroporation and selection of transformants were carried out as previously described [12]. All the strains were grown at 37 °C in Middlebrook 7H9 broth (Difco) supplemented with 0.05% Tween 80 and albumin-dextrose-catalase (ADC) or on solid Middlebrook 7H10 medium (Difco) supplemented with oleic acid-albumin-dextrose-catalase (OADC).

### Chemicals

Thymoquinone (TQ), isoniazid (INH), rifampicin (RIF), and the competitive nitric oxide synthase inhibitor NG-monomethyl-L-arginine (L-NMMA) were obtained from Sigma-Aldrich (USA).

### Cells and culture conditions

Mouse macrophage RAW 264.7 cells were purchased from American Type Culture Collection (ATCC) (USA). Cells were maintained in Dulbecco’s Modified Eagle’s Medium (DMEM) supplemented with 10% (*v*/v) heat-inactivated fetal bovine serum (FBS) and 1% (*v*/v) antibiotic/antimycotic cocktail in a humidified atmosphere of 5% CO_2_ at 37 °C. Cells were seeded (5 × 10^4^ cells/well) in 96-well plates for 24 h.

Type II human alveolar A549 cells were purchased from ATCC (USA). A549 cells were used in this study because of their involvement in tuberculosis infection; MTB can easily invade and replicate within these cells [13] [14], and they also have potential roles in innate immunity and inflammatory responses [15]. Cells were maintained in Dulbecco’s Modified Eagle’s Medium (DMEM/Ham’s F-12) supplemented with 10% (*v*/v) heat-inactivated fetal bovine serum (FBS) and 1% (*v*/v) antibiotic/antimycotic cocktail (100 U/mL penicillin, 100 μg/mL streptomycin, and 0.25 μg/mL amphotericin B; Invitrogen, Carlsbad, CA, USA) in a humidified atmosphere of 5% CO_2_ at 37 °C. Cells were seeded (5 × 10^5^ cells/mL) in 60 × 15 mm tissue culture plates overnight until they reached 75–85% confluence.

Human monocyte THP-1 cells were purchased from ATCC (USA). To investigate the regulation of macrophage-specific genes as they narrate the physiological functions displayed by these cells, we used differentiated THP-1 macrophages because they behave more like native monocyte-derived macrophages [16]. Cells were maintained in Roswell Park Memorial Institute medium (RPMI 1640) supplemented with 4.5 g/L D-Glucose, 2.383 g/L HEPES Buffer, L-Glutamine, 1.5 g/L Sodium Bicarbonate, 110 mg/L Sodium Pyruvate, 10% (*v*/v) heat-inactivated fetal bovine serum (FBS) and 1% (*v*/v) antibiotic/antimycotic cocktail in a humidified atmosphere of 5% CO_2_ at 37 °C. THP-1 monocytes were seeded (1.5 × 10^6^ cells/mL) in 60 × 15 mm tissue culture plates along with 200 nM PMA for 48 h so that they differentiated into macrophages, followed by 24 h of incubation without PMA.

### Infection with M. Tuberculosis

RAW 264.7 cell monolayer was exposed for 2 h to both H37Rv and XDR-TB at a multiplicity of infection (MOI) of 1:1 bacilli per cell in 96-well plates. After removing extracellular bacilli, cells were incubated for an additional 3 days with or without drugs. Cells were lysed with sterile deionized water followed by inoculation on 7H10 agar for CFU (colony forming unit) counting. Alveolar A549 epithelial cells and THP-1 macrophages were exposed for 3 h to H37Rv at a multiplicity of infection (MOI) of 10:1 bacilli per cell in 60 × 15 mm cell culture dishes. After removing extracellular bacilli, cells were incubated for an additional 24 h with or without drugs for further study.

### Cell viability assay

Cell viability was determined by using EZ-Cytox Cell Viability Assay solution WST-1 (Daeil Lab Service, Jong-No, Korea). The assay is based on the conversion of the tetrazolium salt WST-1 into a colored dye by mitochondrial dehydrogenase enzymes. Cells were seeded at a concentration of 7.5 × 10^5^ cells/well for 24 h and co-incubated with drugs for another 24 h. Following incubation, 20 μl of WST-1 solution was added to each well and absorbance was measured at 570 nm using a Victor™ ×3 Multilabel reader (Perkin Elmer 2030) following 3 h of incubation at 37 °C.

### Determination of nitrite accumulation

The concentration of nitrite produced (a means of measuring NO) was measured using Griess reagent (Promega, USA). In brief, supernatants of infected and/or treated cells were collected at specified time points and centrifuged at 400 × g for 8 min to remove cells. In the meantime, nitrite standards were prepared by diluting 100 μM nitrite solution to 0.39 μM by two-fold dilution. Fifty microliters of cell-free supernatants and nitrite standards were added to 96-well tissue culture plates. The assay was done in triplicate. Subsequently, 50 μM sulfanilamide solution was added to each well and incubated at room temperature for 5–10 min followed by the addition of 50 μM N-1-napthylethylenediamine hydrochloride. After 5–10 min, the absorbance was measured at 540 nm using a Victor™ ×3 Multilabel reader (Perkin Elmer 2030).

### Confocal microscopy

Confocal microscopy was performed in H37Ra-GFP infected Raw 264.7 cells as previously described [17]. In brief, Raw 264.7 cells were infected with H37Ra-GFP at an MOI of 1:1 in RPMI 1640 medium supplemented with 10% heat-inactivated FBS for 3 h at 37 °C with 5% CO_2_. After washing twice with pre-warmed PBS, extracellular TB was killed by treatment with amikacin (20 μM) for 1 h. After washing twice, cells were treated with TQ at different concentrations and incubated under cell culture conditions for 5 days. Finally, both the untreated and treated macrophages infected with GFP-TB were stained with Syto60 (5 μM; Invitrogen) for 30 mins at 37 °C and images were acquired using a confocal microscope (Olympus; Tokyo, Japan) and analyzed using FV10i-ASW 3.0 Viewer software.

### Western blot analysis

A549 cells were harvested following infection and/or treatment, and proteins were collected using RIPA lysis buffer containing protease inhibitor cocktail (Santa Cruz Bio-technology, USA). Protein concentration was quantified using a BCA protein assay kit (Pierce, USA) and Western blot analysis was performed as previously described [18].

### qRT-PCR

THP-1 and A549 cells were harvested following infection and/or treatment. Total mRNA was collected, quantified, and checked for purity, cDNA was prepared, and qRT-PCR was performed as previously described [19]. The assay results were normalized to the endogenous control gene GAPDH. The primer pair sequences are listed in Table 1.

**Table 1:** Sequences of primers used for qRT-PCR

Primer Name	Sequence
GAPDH (F)	5′ TCCCATCACCATCTTCCA 3′
GAPDH (R)	5′ CATCACGCCACAGTTTCC 3’
iNOS (F)	5′ ACAAGCTGGCCTCGCTCTGGAAAGA 3’
iNOS (R)	5′ TCCATGCAGACAACCTTGGGGTTGAAG 3’
TNF-α (F)	5′ CAGCCTCTTCTCCTTCCTGAT 3’
TNF-α (R)	5′ GCCAGAGGGCTGATTAGAGA 3’
IL6 (F)	5′ GATGAGTACAAAAGTCCTGATCCA 3’
IL6 (R)	5′ CTGCAGCCACTGGTTCTGT 3’

(*F*) Forward primer, (*R*) Reverse primer, *GAPDH* Glyceraldehyde 3-phosphate dehydrogenase, *iNOS*, Inducible nitric oxide synthase, *TNF- α* Tumor necrosis factor alpha, *IL-6* Interleukin 6

### Statistical analysis

Each experiment was repeated at least three times with negligible differences in the individual results. The statistical significance of the results of different experiments was evaluated using Student’s t-test. Data in graphs are presented as mean ± S.D. Means were considered to be significantly different at a level of *p < 0.05.

## Results

### Influence of TQ on *M. tuberculosis* replication in raw 264.7 cells

#### Colony-forming unit (CFU) assay

**Fig. 1:**
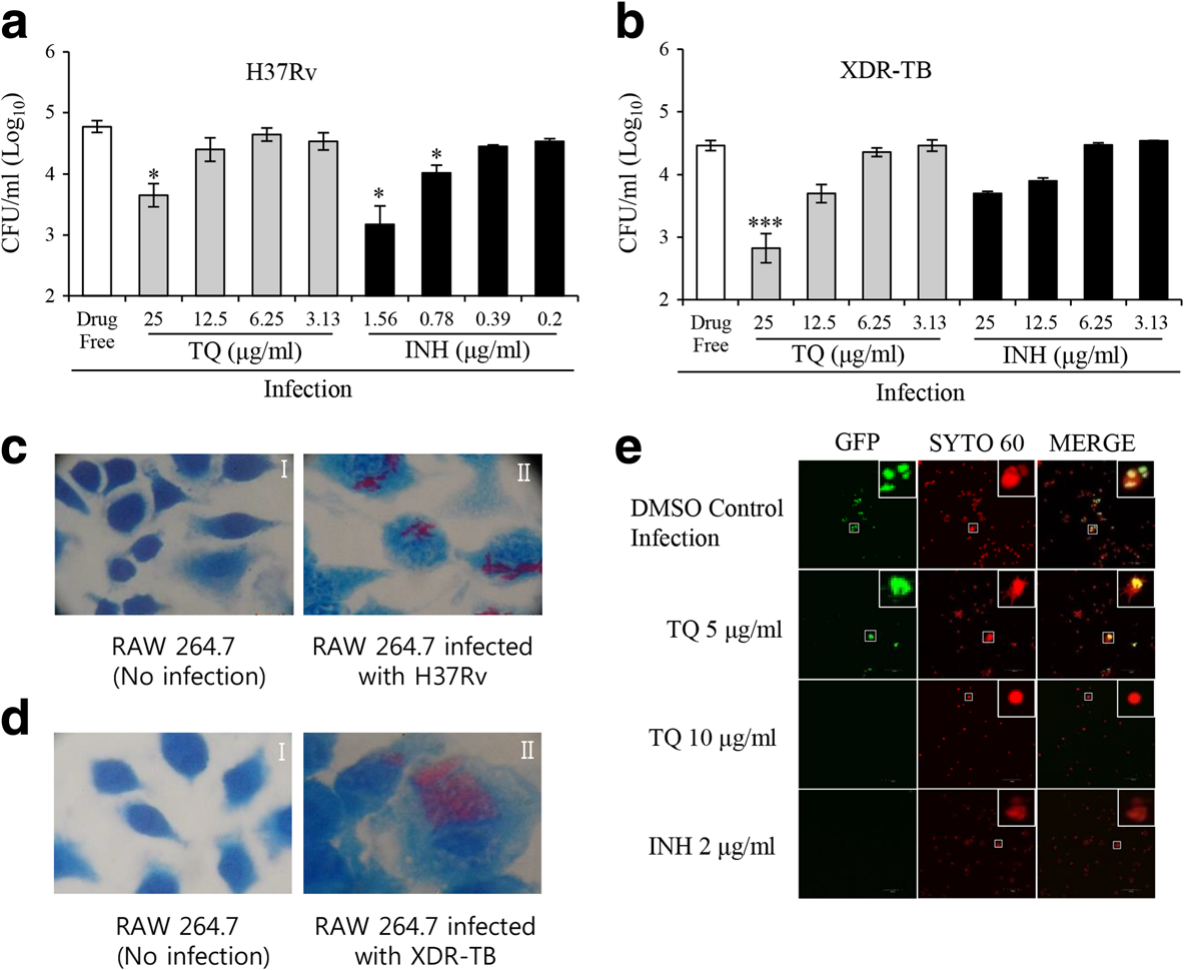
In vitro efficacy data showing the intracellular killing effect of TQ in *M. tuberculosis*-infected macrophages. Raw 264.7 macrophages were infected with H37Rv and XDR-TB for 2 h at an MOI of 1:10 at 37 °C with 5% CO_2_ followed by drug treatment for 3 days. **a** H37Rv burden (log_10_ CFU) in H37Rv-infected macrophages after TQ and INH treatment. **b** XDR-TB burden (log_10_ CFU) in H37Rv-infected cells after TQ and INH treatment. Data represent the mean ± SD of 3 independent experiments done in triplicate. *P < 0.05 by Student’s t-test. In acid-fast staining, methylene blue was used to stain macrophages, while red colored carbol fuchsin stains the bacteria. **c**-*I* Uninfected RAW 264.7 cells. **c**-*II* RAW 264.7 cells infected with H37Rv. **d**-*I* Uninfected RAW 264.7 cells. **d**-*II* RAW 264.7 cells infected with XDR-TB. Raw 264.7 macrophages were infected with GFP-H37Ra for 3 h at an MOI of 1:1 at 37 °C with 5% CO_2_ followed by drug treatment for 5 days. **e** Comparative TQ efficacy in an in vitro model, along with INH

To investigate the effects of TQ on *M*. tuberculosis replication in Raw 264.7 cells, confluent cell layers grown overnight in 96-well plates were infected with H37Rv and XDR-TB at an MOI of 1:1 and treated with TQ at concentrations ranging from 3.13 to 25 μg/mL with the first-line drug isoniazid (INH) in different concentrations according to another experiment (data not shown here). Cells were washed and collected 72 h post-infection (p.i.), and their direct antimicrobial activity on intracellular *M. tuberculosis* H37Rv and XDR-TB were measured by a colony-forming unit (CFU) assay. The number of CFUs was analyzed by harvesting bacteria at 72 h post-infection followed by plating on 7H10 agar plates, where surviving colonies were enumerated as CFU/mL. TQ reduced the titers of *M. tuberculosis* H37Rv and XDR-TB in a concentration-dependent manner (Fig. 1a and b).

TQ significantly reduced the number of viable H37Rv bacilli in a dose-dependent manner after 72 h of incubation, with more than 57% and 92% of the bacteria killed with 12.5 and 25 μg/mL TQ (*p* ≤ 0.05), respectively. On the other hand, 82% and 97% of the intracellular bacteria were killed by 0.78 and 1.56 μg/mL INH (*p* ≤ 0.05), respectively (Fig. 1a).

TQ also reduced the amount of intracellular XDR-TB in a concentration-dependent manner. It showed better efficacy in XDR-TB killing after 72 h of incubation, with more than 82% and 97% of the bacteria being killed with 12.5 and 25 μg/mL TQ (*p* ≤ 0.05), respectively. On the other hand, 72% and 82% of the intracellular bacteria were killed by 12.5 and 25 μg/mL INH, respectively (Fig. 1b).

To assess the infection, acid-fast staining was done. Fig. 1c and d show the infection status compared to without infection for both H37Rv and XDR-TB, respectively.

#### Macrophage infection and image acquisition and analysis

Raw264.7 macrophages were first infected with mycobacteria that constitutively express green fluorescent protein (GFP) at an MOI of 1:1. Infected macrophages were treated with TQ (5 and 10 μg/mL) and INH followed by incubation at 37 °C for 5 days. Confocal images of live samples were acquired using a confocal microscope (Olympus; Tokyo, Japan), and representative results are shown in Fig. 1e. The first column shows GFP-labeled H37Ra, the second column shows syto-60-labeled Raw 264.7, and the third column shows the merged image of both. In comparison with the DMSO (dimethyl sulfoxide) control, samples treated with TQ (5 μg/mL) showed few GFP-H37Ra inside macrophages, but in TQ- (10 μg/mL) and INH-treated samples, the level of green florescence was significantly reduced.

#### Influence of TQ on NO production

**Fig. 2:**
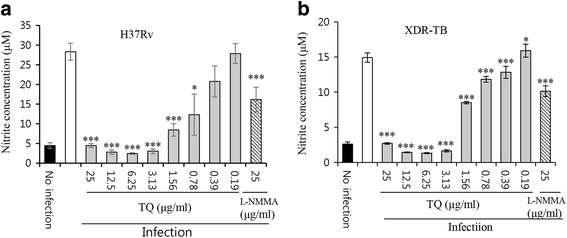
Modulation of MTB-induced NO production by TQ in *M. Tuberculosis*-infected macrophages. Raw 264.7 macrophages were infected with H37Rv and XDR-TB for 2 h at an MOI of 1:10 at 37 °C with 5% CO_2_, followed by drug treatment for 3 days. Nitric oxide (μM) was measured using Griess reagent. **a** Effect of TQ on nitric oxide production in H37Rv-infected Raw 264.7 cells. **b** Effect of TQ on nitric oxide production in XDR-TB-infected Raw 264.7 cells. Data represent the mean ± SD of 3 independent experiments done in triplicate. *P < 0.05, ** < 0.01, *** < 0.009 by Student’s t-test

To investigate the effects of TQ on NO production in MTB-infected Raw 264.7 cells, confluent cell layers grown overnight in 96-well plates were infected with H37Rv and XDR-TB at an MOI of 1:1 and treated with TQ at concentrations ranging from 0.19 to 25 μg/mL. L-NMMA (25 μg/mL), a potent NO synthesis inhibitor, was used as a positive control and NO was measured using Griess reagent. TQ at concentrations ranging from 0.78 to 25 μg/mL significantly reduced NO production in H37Rv-infected macrophages in a dose-dependent manner after 72 h of incubation (*p* ≤ 0.05; Fig. 2a). Furthermore, in XDR-TB-infected macrophages, TQ at concentrations ranging from 0.19 to 25 μg/mL significantly reduced NO production in a dose-dependent manner after 72 h of incubation (*p* ≤ 0.05; Fig. 2b).

#### Influence of TQ on MTB-induced expression of NO and pro-inflammatory cytokines in A549 cells

**Fig. 3:**
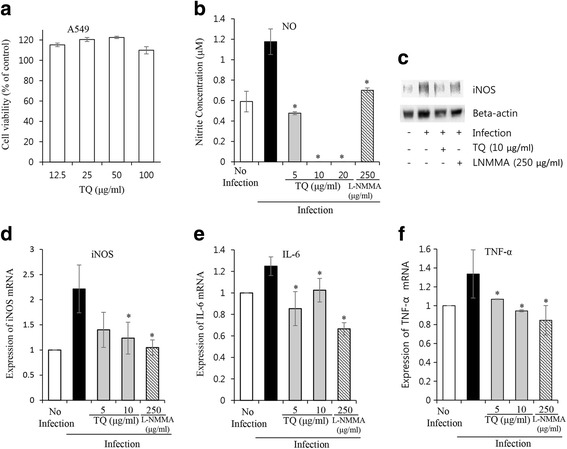
The effect of TQ on MTB-induced NO expression in Type II human alveolar A549 cells. A549 cells were infected with H37Rv for 3 h at an MOI of 1:10 at 37 °C with 5% CO_2_, followed by drug treatment for 24 h. **a** Cytotoxicity of TQ on A549 cells. **b** Effect of TQ on nitric oxide production in H37Rv-infected A549 cells. Data represent the mean ± SD of 3 independent experiments done in triplicate. * *P* < 0.05 by Student’s t-test. **c** Western blot result showing the changes in iNOS protein expression upon TQ treatment; representative result from a triplicate experiment. **d** qRT-PCR result showing the changes in iNOS mRNA expression. **e** Changes in IL-6 mRNA expression. **f** Changes in TNF-α mRNA expression due to TQ treatment. Data represent the mean ± SD of triplicates of an individual experiment and are representative of 3 independent experiments. *P < 0.05 by a two-tailed Student’s t-test

MTB-infected A549 cells were co-cultured for 24 h with varying doses of TQ, and infected/uninfected cultures devoid of TQ treatment served as controls. The cytotoxic effect of TQ (12.5–100 μg/mL) on A549 cells was evaluated using WST reagent, and up to 100 μg/mL was found to be non-cytotoxic in A549 cells (Fig. 3a).

Furthermore, the effect of TQ (5–20 μg/mL) on MTB-induced NO production was measured using Griess reagent, and L-NMMA (250 μg/mL) was used as a positive control. TQ (*p* < 0.05) significantly reduced cellular NO levels in MTB-infected cells (Fig. 3b).

Next, protein expression of the iNOS (inducible nitric oxide) gene was observed following total protein extraction, where β-actin was used as an endogenous loading control. In Western blot data, upregulation of iNOS was observed in H37Rv-infected cells, which was decreased by treatment with TQ (10 μg/mL) (Fig. 3c).

Expression of iNOS mRNA was induced following infection of cells with *M. tuberculosis* H37Rv, which was significantly suppressed by TQ (10 μg/mL) (*p* < 0.05) treatment at the indicated time points, as determined by qRT-PCR (Fig. 3d). Expression of the pro-inflammatory cytokines IL-6 and TNF-α was induced following infection of cells with *M. tuberculosis* H37Rv, which was significantly (*p* < 0.05) suppressed by TQ (5 and 10 μg/mL) treatment at the indicated time points, as determined by qRT-PCR (Fig. 3e and f).

#### Influence of TQ on MTB-induced expression of iNOS and pro-inflammatory cytokines in THP-1 macrophages

**Fig. 4:**
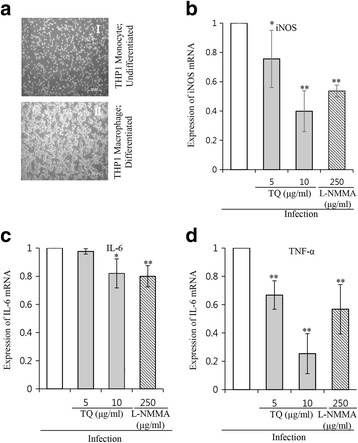
The effect of TQ on MTB-induced NO expression in human monocyte THP-1 cells. THP-1 monocytes were differentiated into macrophages by 200 nM PMA treatment. The cells were imaged at ×10 magnification with an inverted microscope (ZEISS; Axiovert 25). **a**-*I* THP-1 monocytes, undifferentiated. **a**-*II* THP-1 macrophages, differentiated. Cells were infected with H37Rv for 3 h at an MOI of 1:10 at 37 °C with 5% CO_2_, followed by drug treatment for 24 h. **b** q-RT-PCR result showing changes in iNOS mRNA expression. **c** Changes in IL-6 mRNA expression. **d** Changes in TNF-α mRNA expression due to TQ treatment. Data represent the mean ± SD of triplicates of an individual experiment and are representative of 3 independent experiments. *P < 0.05, ** < 0.005 by two-tailed Student’s t-test

THP-1 macrophages were used to reinvestigate the effect of TQ on MTB-induced iNOS and pro-inflammatory cytokine expression seen in A549 cells. THP-1 monocytes were differentiated into macrophages using 200 nM phorbol-12-myristate-13-acetate (PMA; Sigma Chemical Co., St. Louis, Mo) (Fig. 4a). Expression of iNOS and pro-inflammatory cytokines (IL-6 and TNF-α) was induced following infection of cells with *M. tuberculosis* H37Rv, which was significantly (*p* < 0.05) suppressed by TQ (5 and 10 μg/mL) treatment at the indicated time points, as determined by qRT-PCR (Fig. 4b-4d).

## Discussion

Approximately 3 people in the world die of tuberculosis every minute [20]. Infection caused by antibiotic-susceptible pulmonary TB can readily be managed with the first-line drugs INH and RIF, but infections caused by MDR and XDR tuberculosis strains are difficult to deal with using first-line drugs despite the increasing use of second-line drugs [21]. Furthermore, the emergence of MDR and XDR tuberculosis with added resistance to all fluoroquinolones and other antituberculosis drugs, such as kanamycin, amikacin, and capreomycin, has been reported by 84 countries from all around the world [22]. This trend necessitates a new therapeutic option with better efficacy to combat tuberculosis, which led our present efforts to investigate the potential role of TQ as an anti-tuberculosis drug. TQ has been reported as a potential anti-tuberculosis drug against both drug-susceptible and drug-resistant *M. tuberculosis* [8], and the therapeutic concentration of TQ used in this study was selected accordingly. As *M. tuberculosis* is phagocytosed by macrophages in the lungs, which are thought to be the predominant host cells for the majority of its infectious life cycle [20], we focused on the intracellular killing effect of TQ in macrophages, which has not yet been elucidated. But localization of the pathogen is the most critical point of antibiotic efficacy in intracellular killing [23]. As the intracellular localization of pathogen protects it from some antibiotics, and this fact must be taken into account to develop new anti-bacterial compounds [24]. In case of TQ, it has been reported that thymoquinone can potentially inhibit the replication of intracellular *Candida albicans* [25] and intracellular Epstein–Barr virus (EBV) [26], which proves the efficacy of TQ to invade the cell and make a possible candidate to kill the intracellular *Mycobacterium tuberculosis*.

*Mycobacterium tuberculosis* infection can induce the production of IL-6 [27] and TNF-α [28]. MTB infection also induce nitric oxide (NO) expression by inducible nitric oxide synthase (iNOS) [4]. An abundance of different cytokines, including IL-6 and TNF-α, can lead to inflammation, which can in turn lead to several diseases, including Alzheimer’s disease [29], cardiovascular diseases [30], inflammatory bowel disease [31], sickness behavior [32], tumor progression [33], and so on. Apart from the role of TNF-α in controlling *Mycobacterium tuberculosis* infection, it can also cause severe tissue damage [34].

Furthermore, host-derived stress factors such as NO or other pro-inflammatory cytokines can induce drug tolerance [7]. Interestingly, TQ was reported as a potential anti-inflammatory drug in different experimental settings [10] [11] [35], and has the ability to reduce the production of cellular nitric oxide [36]. Furthermore, TQ decreases both the serum and pancreatic nitrite concentration in streptozotocin (STZ) rat diabetic model [37], it also significantly decreases the kidney and liver nitrite and TNF-α level in methotrexate-(MTX) treated rat [38]. In animal model of arthritis also showed that thymoquinone could significantly reduce serum nitric oxide, urea and creatinine levels and eventually prevent kidney dysfunction [39]. Therefore, we investigated the role of TQ in the reduction of MTB-induced NO and expression of other cytokines such as IL-6 and TNF-α.

## Conclusions

To the best of our knowledge, the intracellular killing ability of TQ in MTB-infected macrophage have been demonstrated for the first time in this study. Our study showed that TQ successfully inhibits the replication of *M. tuberculosis* H37Rv and XDR-TB inside Raw 264.7 macrophage and also inhibits the production of NO and other cytokines in different cell lines. Based on this reports, we believe that further in-depth studies are warranted to explore the effect of thymoquinone treatment in in-vivo model to validate the in vitro effect. Furthermore, future studies should be undertaken to illustrate the exact mechanism of action of TQ as an anti-mycobacterial agent.

## Abbreviations

A549: Human type II alveolar epithelial cells
ADC: Albumin-dextrose-catalase
ATCC: American Type Culture Collection
CFU: Colony forming unit
DMEM: Dulbecco’s Modified Eagle’s Medium
DMSO: Dimethyl sulfoxide
FBS: Fetal bovine serum
GAPDH: Glyceraldehyde 3-phosphate dehydrogenase
GFP: Green fluorescent protein
IL-6: Interlukin-6
INH: Isoniazid
INOS: Inducible nitric oxide synthase
L-NMMA: NG-monomethyl-L-arginine
MDR-TB: Multidrug-resistant tuberculosis
MOI: Multiplicity of infection
MTB: *Mycobacterium tuberculosis*
NO: Nitric oxide
NOS: Nitric oxide synthase
OADC: Oleic acid-albumin-dextrose-catalase
PMA: Phorbol-12-myristate-13-acetate
RIF: Rifampicin
RNI: Reactive nitrogen intermediate
ROS: Reactive oxygen species
RPMI: Roswell Park Memorial Institute medium
TNF-α: Tumor necrosis factor-alpha
TQ: Thymoquinone
XDR-TB: Extensively drug-resistant tuberculosis
